# Intraoperative Electrocochleographic Characteristics of Auditory Neuropathy Spectrum Disorder in Cochlear Implant Subjects

**DOI:** 10.3389/fnins.2017.00416

**Published:** 2017-07-19

**Authors:** William J. Riggs, Joseph P. Roche, Christopher K. Giardina, Michael S. Harris, Zachary J. Bastian, Tatyana E. Fontenot, Craig A. Buchman, Kevin D. Brown, Oliver F. Adunka, Douglas C. Fitzpatrick

**Affiliations:** ^1^Department of Otolaryngology/Head and Neck Surgery, Ohio State University College of Medicine Columbus, OH, United States; ^2^Lab Department of Otolaryngology/Head and Neck Surgery, University of Wisconsin School of Medicine Madison, WI, United States; ^3^Department of Otolaryngology/Head and Neck Surgery, The University of North Carolina at Chapel Hill School of Medicine Chapel Hill, NC, United States; ^4^Department of Otolaryngology/Head and Neck Surgery, Washington University School of Medicine in St. Louis St. Louis, MO, United States

**Keywords:** cochlear implants, electrocochleography, auditory neuropathy spectrum disorder, intraoperative, pediatrics, cochlear microphonic

## Abstract

Auditory neuropathy spectrum disorder (ANSD) is characterized by an apparent discrepancy between measures of cochlear and neural function based on auditory brainstem response (ABR) testing. Clinical indicators of ANSD are a present cochlear microphonic (CM) with small or absent wave V. Many identified ANSD patients have speech impairment severe enough that cochlear implantation (CI) is indicated. To better understand the cochleae identified with ANSD that lead to a CI, we performed intraoperative round window electrocochleography (ECochG) to tone bursts in children (*n* = 167) and adults (*n* = 163). Magnitudes of the responses to tones of different frequencies were summed to measure the “total response” (ECochG-TR), a metric often dominated by hair cell activity, and auditory nerve activity was estimated visually from the compound action potential (CAP) and auditory nerve neurophonic (ANN) as a ranked “Nerve Score”. Subjects identified as ANSD (45 ears in children, 3 in adults) had higher values of ECochG-TR than adult and pediatric subjects also receiving CIs not identified as ANSD. However, nerve scores of the ANSD group were similar to the other cohorts, although dominated by the ANN to low frequencies more than in the non-ANSD groups. To high frequencies, the common morphology of ANSD cases was a large CM and summating potential, and small or absent CAP. Common morphologies in other groups were either only a CM, or a combination of CM and CAP. These results indicate that responses to high frequencies, derived primarily from hair cells, are the main source of the CM used to evaluate ANSD in the clinical setting. However, the clinical tests do not capture the wide range of neural activity seen to low frequency sounds.

## Introduction

Auditory neuropathy spectrum disorder (ANSD) is a hearing dysfunction characterized by an apparent discrepancy between the measures of cochlear and neural function when viewed by surface electrode auditory brainstem response (ABR) testing. Relatively healthy hair cells are identified by the presence of otoacoustic emissions (OAEs) and/or cochlear microphonic (CM) in ABR testing, coupled with small or absent wave V (Kaga et al., 1996; Starr et al., 1996; Berlin et al., 1998; Rance et al., 1999; Teagle et al., 2010). A wide range of etiologies and associations for ANSD has been identified, including perinatal hyperbilirubinemia, mechanical ventilation, infection (measles, mumps), mutations in the otoferlin gene and cochlear nerve deficiency (Starr et al., 2001; Varga et al., 2003; Buchman et al., 2006; Bielecki et al., 2012). Proposed sites of lesion include the inner hair cells (IHCs), the synapse between the IHCs and the type I afferents of the auditory nerve, the auditory nerve itself, and the synapse between the auditory nerve fibers and their targets in the cochlear nucleus (Starr et al., 1996; Doyle et al., 1998; Zeng et al., 1999; Berlin et al., 2003; Fuchs et al., 2003; Rapin and Gravel, 2003). Many subjects with ANSD have hearing loss and/or speech perception deficits severe enough that treatment with a cochlear implant (CI) is indicated. A number of studies of the electrocochleography (ECochG) of ANSD subjects receiving CIs have been done, however these studies used acoustic stimuli specialized for this group such as high frequency 8 kHz tone pips or clicks (McMahon et al., 2008; Santarelli et al., 2008; Santarelli, 2010; Stuermer et al., 2015). While high frequencies may be useful in diagnosis, most of the ECochG responses in CI subjects, in both children and adults, are in fact to low frequencies (Fitzpatrick et al., 2014; McClellan et al., 2014; Formeister et al., 2015). Thus, to compare ANSD with non-ANSD subjects, responses to both high and low frequencies must be obtained. For this study, we recorded responses to tones across the frequency range in CI subjects, both children and adults, with and without ANSD.

Speech perception outcomes with cochlear implantation, including those with ANSD, demonstrate wide variations from patient to patient (Cohen et al., 1991; Gantz et al., 1993; Firszt et al., 2004; Holden et al., 2013). Most studies have failed to demonstrate specific factors or combinations of factors that account for more than about 25% of the variance in outcomes (Shea et al., 1990; Fayad et al., 1991, 2006; Gantz et al., 1993; Shipp and Nedzelski, 1995; Blamey, 1997; Nadol, 1997; Shipp et al., 1997; Rubinstein et al., 1999; Friedland et al., 2003; Lazard et al., 2012; Blamey et al., 2013). A recent measure used in both adults and children is the “total response” seen in the ECochG responses (ECochG-TR), which is the sum of the spectral peaks in response to tones of different frequencies. In adults, the ECochG-TR has been shown to account for about 40–50% of the variance in speech perception outcomes (Fitzpatrick et al., 2014; McClellan et al., 2014). In a specific group of children old enough for word test scores to be administered, the ECochG-TR accounted for 32% of the variance (Formeister et al., 2015). Thus, ECochG-TR provides a description of residual cochlear physiology that could prove useful in providing counseling and rehabilitation on the basis of patient-specific factors.

When using low frequency tones, the “on-going response” (continuous steady state response to tones) of the ECochG signal, which is used to calculate the ECochG-TR, is typically composed of the cochlear microphonic (CM) and the auditory nerve neurophonic (ANN). The CM is derived from currents through mechano-sensitive transduction channels in the stereocilia of hair cells (Dallos, 1973), and the ANN is the evoked potential correlate of phase-locking in auditory nerve fibers (Snyder and Schreiner, 1984; Henry, 1995). It is similar to the frequency-following response recorded from the scalp, except that the phase-locking represented is dominated by the auditory nerve rather that brainstem sources. Potentials more commonly seen to high frequencies include the compound action potential (CAP) and summating potential (SP). The CAP represents synchronous firing of auditory nerve fibers to the onsets of sounds, and the SP is derived from complex mixture of sources that roughly follows the envelope, which to tones is a sustained baseline offset. In short, the CM is a hair cell potential, the ANN and CAP are neural potentials, and the SP is affected by hair cell and neural sources capable of envelope-following. Unfortunately, methods to quantify the contributions of the different sources to each potential are lacking, particularly in CI subjects. The major contributor to the TR is the CM, but to low frequencies in many cases the ANN is also present. Although, the presence of the ANN affects the patterns of distortions and spectral components in the recording, a quantitative separation is not currently available. In addition, the morphology of the CAP in CI subjects is highly variable (Scott et al., 2016), and to low frequencies it is mixed with the CM while to high frequencies it can be mixed with the SP, so quantification is difficult. Thus, there is at present no method for determining the proportion of the ECochG that can be considered neural. However, the presence and to an approximation the strength of the ANN and CAP are visually apparent in the recordings, so the approach used here was to score these components individually and add the results to produce a “nerve score” in each case. The CM and SP to high frequencies were also measured as clues to the relative contributions of hair cells to the ECochG.

## Methods

Data in this study include 296 ears from 267 subjects (29 were second sides). Of these, 285 ears were studied under the approval of the Institutional Review Board (IRB) at the University of North Carolina at Chapel Hill (#05-2616) and 11 ears from the Ohio State University (OSU) and Nationwide Children's Hospital (Ohio State University IRB approval #2015H0045). Adults and pediatric (<18 years of age) CI recipients who were English speaking or whose parents were English speaking, and whose ear for implantation was not atretic, were offered enrollment in the study. Written informed consent was obtained from all adults, and parental/guardian consent was obtained for all pediatric subjects. Children who had attained 7 years of age were also asked to assent to participate in the study. In the situation where both ears were implanted and recorded, each ear was considered separately.

### ANSD subjects

A total of 48 ears (39 subjects) were in the ANSD group, 45 ears from children and 3 from adults. The evaluation and management paradigm for children with ANSD is the same between participating study institutions, which for UNC has been published previously (Buchman et al., 2006; Roche et al., 2010; Teagle et al., 2010; Hang et al., 2012). The diagnosis of ANSD was established by the finding of absent or disordered auditory neural activity in the setting of preserved cochlear function, typically established with ABR and OAE testing. Preserved cochlear function was determined when OAEs were present or the early part of the ABR waveforms demonstrated reversal of polarity with alternation of the stimulus polarity in either click or pure tone testing- representing a present CM. Most children were diagnosed with ANSD in our tertiary institutions though some were referred for treatment after a diagnosis was established. All available diagnostic tests were reviewed to confirm the electrophysiological phenotype and diagnosis. The adults all underwent routine CI evaluation, and were tested with a “click” ABR to confirm CM presence. Other groups used for comparison included children (119 ears, 101 subjects) and adults (163 ears, 158 subjects) undergoing cochlear implantation who were not classified as having ANSD.

### Surgical and recording setup

All ECochG recordings were made to acoustic stimulation from the round window (RW) intraoperatively during CI surgery. For the purposes of this study, a foam insert earphone was placed and secured in a manner to prevent occlusion of the sound tubing. The inverting and common electrodes were placed behind the contralateral mastoid and on the glabella, respectively. A standard transmastoid facial recess approach was employed. The anterosuperior portion of the RW overhang was drilled to provide better access to the RW niche. A monopolar electrode (Neurosign, Magstim Co., Wales, UK or Neuro-Kartush raspatory probe instrument, Integra, Plainsboro, NJ, U.S.A.) was then placed with the tip situated immediately within the RW niche. Impedance of the RW and surface electrodes were measured and recordings were terminated if any had impedances of greater than 16 kilo-ohms (kΩ) that could not be reduced below this point. Saline was introduced into the RW niche if the monopolar electrode impedance was high; this was typically enough to bring the impedance measurement to an acceptable level. The Bio-logic Navigator Pro (Natus Medical Inc., San Carlos, CA) system was used to generate acoustic stimuli and record responses. Acoustic stimuli were delivered from Etymotic speaker (ER-3b) through sound tubing and insert earphones. Responses to a frequency series were performed in all subjects, and in most subjects a level series was then performed at the frequency which elicited the strongest response during the prior sweep (typically 500 Hz). The frequency series consisted of 250, 500, 750, 1,000, 2,000, and 4,000 Hz tone bursts presented in alternating phase at 90 dB nHL (101–112 dB SPL). A Blackman window was used to shape the tone bursts which had 1–4 ms rise and fall times with plateaus ranging from 5 to 20 ms (lower frequencies 250–750 Hz had shorter rise and fall times with longer plateaus compared to higher frequencies). Next the level series began at 90 dB nHL and was typically performed in 10 dB decrements until no response was seen during the recordings. Condensation, rarefaction, as well as the difference and sums of pairs of these were stored as averages in separate buffers. A final trial was included where the sound tubing was occluded with a surgical clamp to ensure the recorded responses were not speaker artifact.

### Physiologic analysis

The ECochG results were processed and analyzed using custom software routines written in MATLAB. The condensation and rarefaction traces were extracted and used to calculate the sum and difference waveforms. To evaluate the overall residual response magnitude from each cochlea we measured the “total response,” or ECochG-TR, from the ongoing, steady-state part of the response to the tones (Fitzpatrick et al., 2014). To estimate the proportion of the neural as opposed to hair cell activity we developed a “nerve score” based on visual analysis of the CAP and ANN.

### ECochG-TR

For each frequency a window (4–12 cycles per window dependent on frequency with bin widths ranging from 62 Hz at 250–331 Hz at 4,000 Hz) that isolated the ongoing portion, which occurs after the CAP and prior to the end of the stimulus, was selected for a fast Fourier transform (FFT) to analyze the spectral characteristics of the response. A significant response at a given stimulus frequency or harmonic was present if it exceeded the noise level by three standard deviations. The noise and its variance were determined from up to 6 bins, 3 on each side of the peak that were outside the ranges of response to the stimulus frequency. Responses that were not significant were given a value of 0.02 μV, which is the limit of our detection threshold, when included in summary data. The ECochG-TR was calculated as the sum of the magnitudes of the significant responses at the first, second and third harmonics across all 6 stimulus frequencies, all presented at 90 dB nHL. The first and third harmonics were measured from the difference of the two phases, and the second harmonic from the sum.

### Nerve score

**Figure 1:**
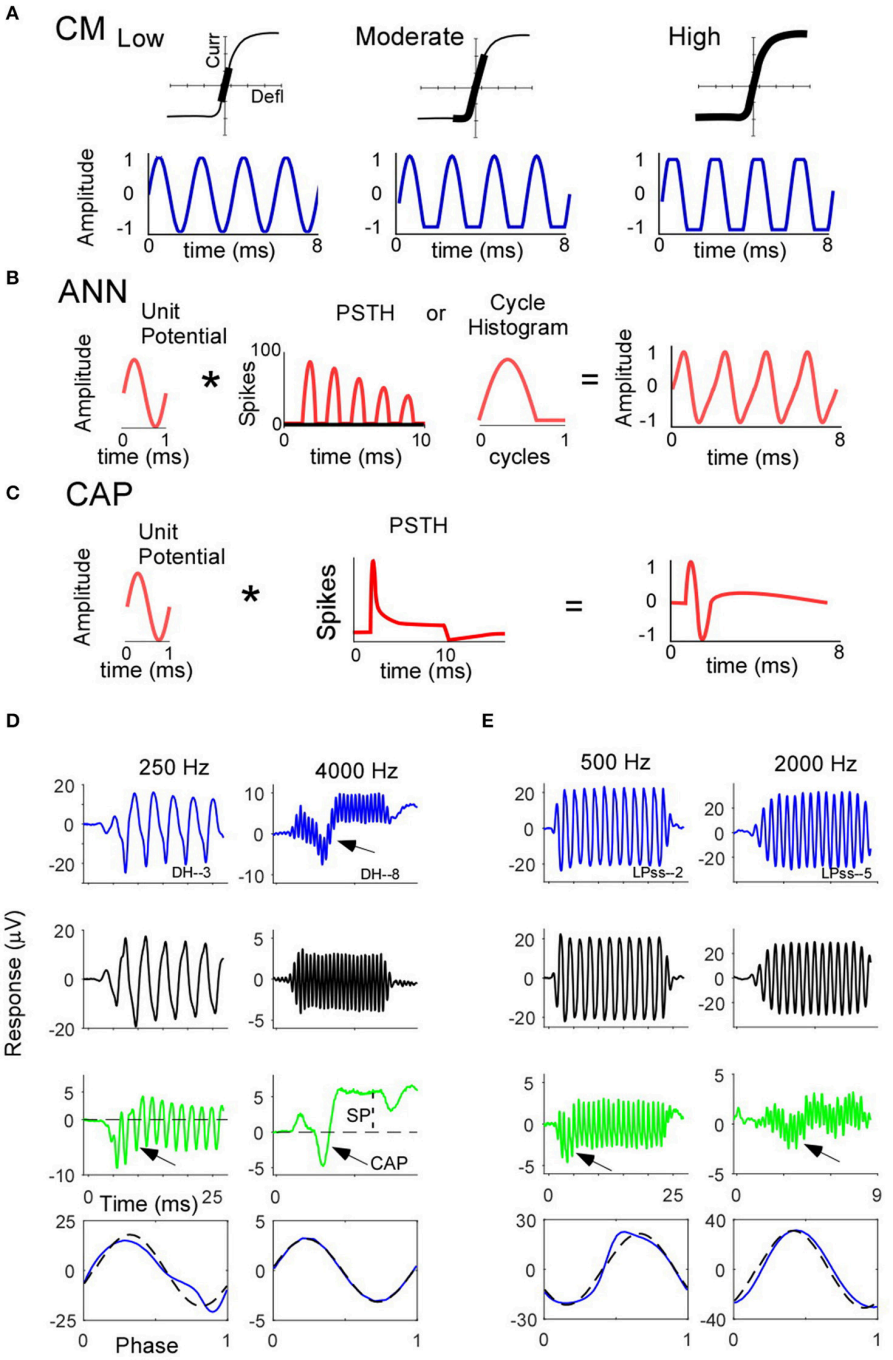
Schematics of the sources of the CM, ANN, and CAP **(A–C)** and examples of ECochG responses obtained from two CI subjects **(D,E)**. **(A)** Typical input-output function of hair cell transduction (top row) producing asymmetries in saturation points as a function of intensity (bottom row). **(B)** The ANN is produced by the convolution (^*^) of a unit potential, or shape of an action potential as it appears at the round window, and the cyclic response to a low frequency in the population of unit responses, which is equivalent to the cycle histogram. The waveform expected is shown to the right. **(C)** The CAP is produced by the convolution (^*^) of the unit potential and well-timed onset responses in the population. **(D,E)** Responses from two subjects to a low and a high frequency tones. For each subject and frequency, the first three rows are, respectively, the responses to condensation phase of stimulation, the difference between the responses to condensation and rarefaction phases (not shown), and the sum of the responses to the two phases. The fourth row depicts an “average cycle” which is the average of all cycles from condensation phase stimuli in a window after the CAP, and from rarefaction stimuli after flipping and shifting the response in time to match that of the condensation phase. See text for further explanation of features identified in these examples.

In the Introduction, we described some of the issues related to measuring and separating the different potentials in the ECochG. Here, we will describe the presumed sources for each potential and describe in more detail the issues with more quantitative measurements that lead to the development of the nerve score. In Figures 1A–C we show schematics of the sources of the CM, ANN and CAP, respectively. The CM is derived from the opening and closing of transduction channels in the stereocilia of hair cells that follows the sinusoidal motion of the basilar membrane. However, the input/output function of each hair cell has limits based on saturation of channel openings or closings, so the CM is only nearly sinusoidal to low intensities (Figure 1A; see Russell, 2008 for a review). In addition, the saturation in the two directions of motion can be asymmetric, with the operating point, or proportion of channels open at rest, typically <50%. Thus, to moderate intensities there will be asymmetric saturation, and then to high intensities there will be a symmetric component as saturation occurs to both directions of motion. The distortions produced by these limits, in the absence of higher order features such as adaptation, would be expected to produce a flattening of the peaks in the ECochG from the CM. Spectrally, the asymmetric component of the saturation produces even harmonics of the fundamental, including zero or DC, while the symmetric component produces odd harmonics (Teich et al., 1989). For moderate and high intensities the population recording will be from regions with various degrees of saturation as the excitation spreads basally. The function shown is generic to illustrate these basic points; *in vivo* IHCs are thought to have more asymmetric input/output functions than OHCs, and basal IHCs are more asymmetric than apical. In recordings from the round window in a noise-damaged cochlea the degree of hair cell asymmetry contributing to the population response is difficult to predict.

The ANN is the evoked potential correlate of neural phase-locking to low frequency stimuli, which is the firing of action potentials over restricted portions of a stimulus cycle. Like the CAP (Goldstein and Kiang, 1958; Wang, 1979; Chertoff, 2004), the ANN can be considered to be the result of a convolution of a unit potential with the post-stimulus time histogram (PSTH) from the population of neural responses (Figure 1B). The unit potential is the representation of a single action potential observed at the round window, which has been described using spike-triggered averaging (Kiang et al., 1976; Prijs, 1986; Versnel et al., 1992). To low frequencies, the PSTH of single neurons contains peaks separated by the stimulus period, which can be folded into the cycle histogram (Rose et al., 1967; Johnson, 1980; Palmer and Russell, 1986). The cycle histogram shows rectification since the firing rate cannot go below zero. The population cycle histogram is less well-understood, but would presumably include some smearing in time and phase when averaged across multiple fibers. The smearing must be relatively small, because the ANN is a prominent feature of the responses to low frequency sounds (Snyder and Schreiner, 1984; Henry, 1995; Forgues et al., 2014; Lichtenhan et al., 2014; Verschooten et al., 2015).

As mentioned, the CAP is produced by the convolution of the unit potential and the population PSTH of auditory nerve fibers (Figure 1C). The CAP is a prominent feature because of the synchronous firing of action potentials that occur to the onset of sounds. These onset responses are timed most precisely to broad band stimuli produced by fast rise times. Thus, the CAP is stronger to high than to low frequency sounds, where the rise time is limited by the stimulus period.

The SP (not shown) is produced by multiple sources capable of producing a DC response to tones. These include the asymmetry in hair cell transduction, which is likely to different between inner and outer hair cells, which also differ in their membrane properties (Kros, 2007). The auditory nerve has also been shown to contribute to the SP in several studies (van Emst et al., 1995; Sellick et al., 2003; Forgues et al., 2014). For the auditory nerve, the DC is unlikely to be due to timing in PST, which, unlike the CAP and ANN is asynchronous to high frequencies and intensities. However, an asymmetry in the unit potential, even if small, could produce a DC given the large number of action potentials produced in response to even moderate levels of sound. Such asymmetry in the unit potential has not yet been shown.

With these features of the ECochG signal in mind, the goal of this study is to subjectively characterize the presence of neural compared to hair cells components in the responses to tones of children with and without ANSD who are receiving CIs. The neural components are the ANN and the CAP, with some neural contribution to the SP a possibility as well. The descriptions of the sources of these potential provided above helps to explain why the ANN and CAP are difficult to quantify, such that only a qualitative method was used. That is, the ANN is always mixed with the CM in the ongoing part of the response to the low frequency tone. However, the ANN is generally the more distorted signal, because the shape of the unit potential is unrelated to the stimulus, and the cycle histogram is roughly half-wave rectified. So the presence of strong harmonics, both even and odd, is evidence of the presence of the ANN. However, due to its periodicity the ANN's magnitude cannot be known because some or most will be in the first harmonic, where the largest part of the CM also resides. Furthermore, some of the CM can be in the higher harmonics due to the asymmetric and symmetric distortions described in Figure 1A, so the simple presence of higher harmonics of either even or odd order is not proof that the ANN is present. Thus, there is no simple method to objectively identify or quantify the CM and ANN contributions to the ongoing response.

In many CI subjects the CAP is obvious and can be measured using accepted methods. However, in a recent study (Scott et al., 2016) only 50% of CI subjects showed a CAP, and the difficulties in measurements were described, including (1) most CI subjects have responses only to low frequencies where the CAP is small compared to high frequencies; (2) the frequency content of the CAP, centered near 1,000 Hz, overlaps that of the stimulus frequencies that produce the largest responses in CI subjects, preventing the use of filtering to separating the CAP and CM, and (3) some CI subjects have an SP that is strong and rising (or falling) relative to the CAP, so that determining the strength of the CAP is problematic even when one is visually apparent.

The SP is relatively easily quantified as a sustained shift in the baseline. Here the problem is one of interpretation, since the sources of the SP are less than fully clear. However, we will present data from ANSD subjects that suggests there is a neural contribution to the SP.

For these reasons we have not yet found an acceptable objective means of identifying neural activity in each case. That is, although many features, such as large CAP or large harmonic distortions clearly correlate with neural activity, each metric has issues with false positive or negatives. In most cases, the reasons for the results can be observed in the responses themselves when further examined. In Figures 1D,E, we present examples of the data subsequently used to determine the “nerve score,” based on the presence and approximate strength of the ANN and CAP. For each case the responses to a low and to a high frequency are presented, with low defined as in the range of strong phase-locking and high as above that range as defined in animal studies (Weiss and Rose, 1988). The first three rows in the figures are, respectively, the responses to condensation phase of stimulation (top), the difference between the responses to condensation and rarefaction phases (second row), and the sum of the responses to the two phases (third row). The responses to the condensation phase stimulus are the “raw” data, while the difference curves represent the part of the responses that changes with the change of polarity of the stimulus, and the summed curves represents the parts of the responses that don't change with the stimulus phase. These features make the difference curve contain predominantly odd-order harmonics, dominated by the first harmonic at the stimulus frequency, while the summed curve contains predominantly the even-order harmonics, particularly the second which is at twice the stimulus frequency. The CAP (arrows) is usually most visible in the summed curve when present, as is the SP. The bottom row shows the “average cycle” obtained from the cycles of response after the CAP and prior to the stimulus offset. This average cycle is where the distinct types of distortions characteristic of the CM and ANN can best be seen.

The example in D is a case with both a strong ANN and a strong CAP. A strong ANN is suggested by the prominent response at twice the stimulus frequency seen in the summed curve to the 250 Hz stimulus, and is clearly seen in the “average cycle” (lower left, solid curve). This curve is the average of all cycles from condensation phase stimuli in a window after the CAP, and from rarefaction stimuli after shifting the response in time to match that of the condensation phase. The average curve in this case is highly distorted compared to a sinusoid representing the stimulus (dashed line) that has been shifted in phase to have the best fit to the response. The lack of an ANN to the high frequency stimulus is shown by the lack of an AC component in the summed curve, and to a purely sinusoidal average cycle. The CAP (arrows) is most clearly seen to the high frequency stimulus in the summed curve, but is also readily visible in the response to condensation phase stimuli. However, it is embedded in an SP that is rising during the same time period, making its measurement problematic.

The example in E is a case where the ANN and CAP were small relative to the CM. To the low frequency stimulus there was still an AC component in the summed curve, but inspection of the average cycle showed a peak-flattened shape that is consistent with rectification of the CM as much as the presence of an ANN. There is also some AC response to twice the stimulus frequency in the summed curve to the high frequency stimulus, representing asymmetry in the CM rather than the ANN. To both frequencies a CAP is present but small CAP (arrows).

Because of these difficulties in measurement of the ANN and CAP we devised a subjective scale termed the “nerve score.” To classify the presence and strength of the ANN we examine the average cycle of the ongoing response to low frequency tones (1,000 Hz and less). An ANN was considered present when the response appeared as a distorted version of a sinusoid, and the distortion was not compatible with a simple rectification or saturation of the CM. Our previous animal experiments where the neural responses were removed with kainic acid (Forgues et al., 2014) demonstrated that removing the neural activity removes these complex distortions, but leaves the peak-flattening type typical of the CM. The CAP was identified primarily in the summed curves, but in some responses to low frequencies the CAP shifts when the phase is changed by a time interval similar to the stimulus period, such that it shows up either exclusively or partially in the difference curve. The strength of *each neural potential* was defined on a scale of 0–2, where a score of 0 indicated there was no identifiable neural contribution across any frequency, 1 indicated small but clear evidence for the component and 2 indicated a large CAP or ANN to one or more frequencies. Once the CAP and ANN were individually scored, *their scores were then added* to produce a nerve score, with a range of 0 (no CAP or ANN) to 4 (CAP and ANN both strong). The case in D was given a nerve score of 4 because both the ANN and CAP were 2's, while the case in E had a nerve score of 1 because of the small but CAP but no definitive evidence of an ANN. Additional examples of data leading to particular nerve scores are provided in the results.

### The SP

To tones, the SP is a baseline shift that persists for the duration of the tone (Figure 1D). Using the summed curve, this shift was measured by averaging points in the 2 ms prior to stimulus onset (i.e., the baseline) and offset (i.e., during the response), and computing the difference.

## Results

### ECochG-TR

**Figure 2:**
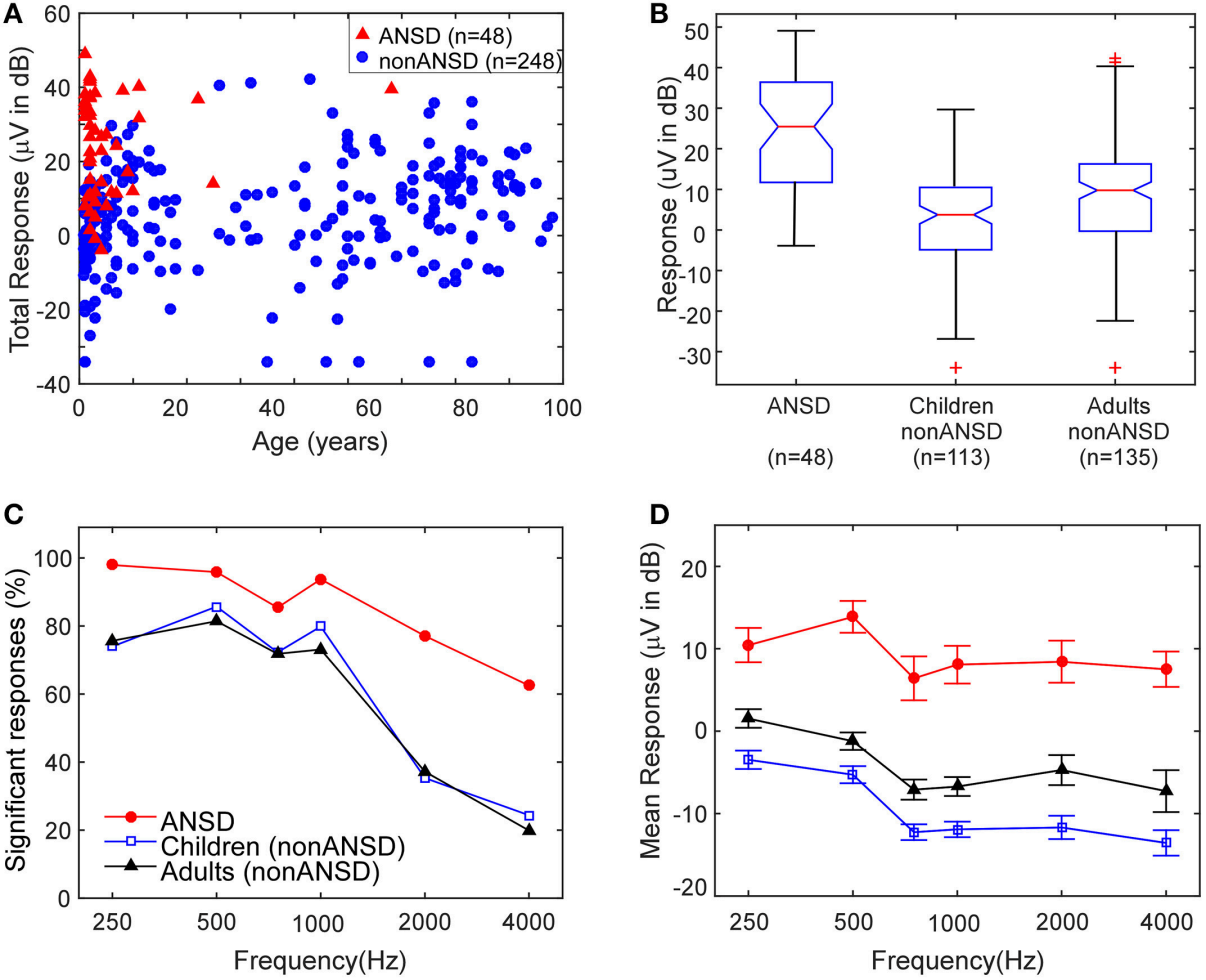
RW ECochG-TR for all CI ears. **(A)** Scatter plot of ECochG-TR vs. Age. **(B)** Distributions of ECochG-TR for three groups. **(C)** Percent of cases with significant responses across frequency for the three groups. **(D)** Magnitude of significant responses across frequency for the three groups.

The ECochG-TR magnitudes as a function of age for the entire cohort are depicted in Figure 2A. The cases with ANSD (Figure 2A, triangles) were found at the upper end of the magnitude distribution. The overall distributions of ECochG-TR for the ANSD subjects, non-ANSD children, and non-ASND adults are shown in Figure 2B. The ANSD cohort had the highest median ECochG-TR, followed by the adults and the non-ANSD children. The differences were significant both as a main effect of group (Kruskal–Wallis, df = 2, chi-sq = 61.1, *p* < 0.0001), and for each comparison (*p* < 0.01).

The proportion of significant responses obtained at each frequency was also different among the groups (Figure 2C). For frequencies of 1,000 Hz and lower, the proportions of ears with significant responses were nearly universal for ANSD cases, and were ~80% of ears in the other groups. Above 1,000 Hz, the proportion of ears with responses declined in all groups, but ANSD subjects had a smaller decline. When present, the magnitudes of the responses (Figure 2D) were higher to all frequencies in the ANSD ears compared to the others.

### Pediatric cohort

**Figure 3:**
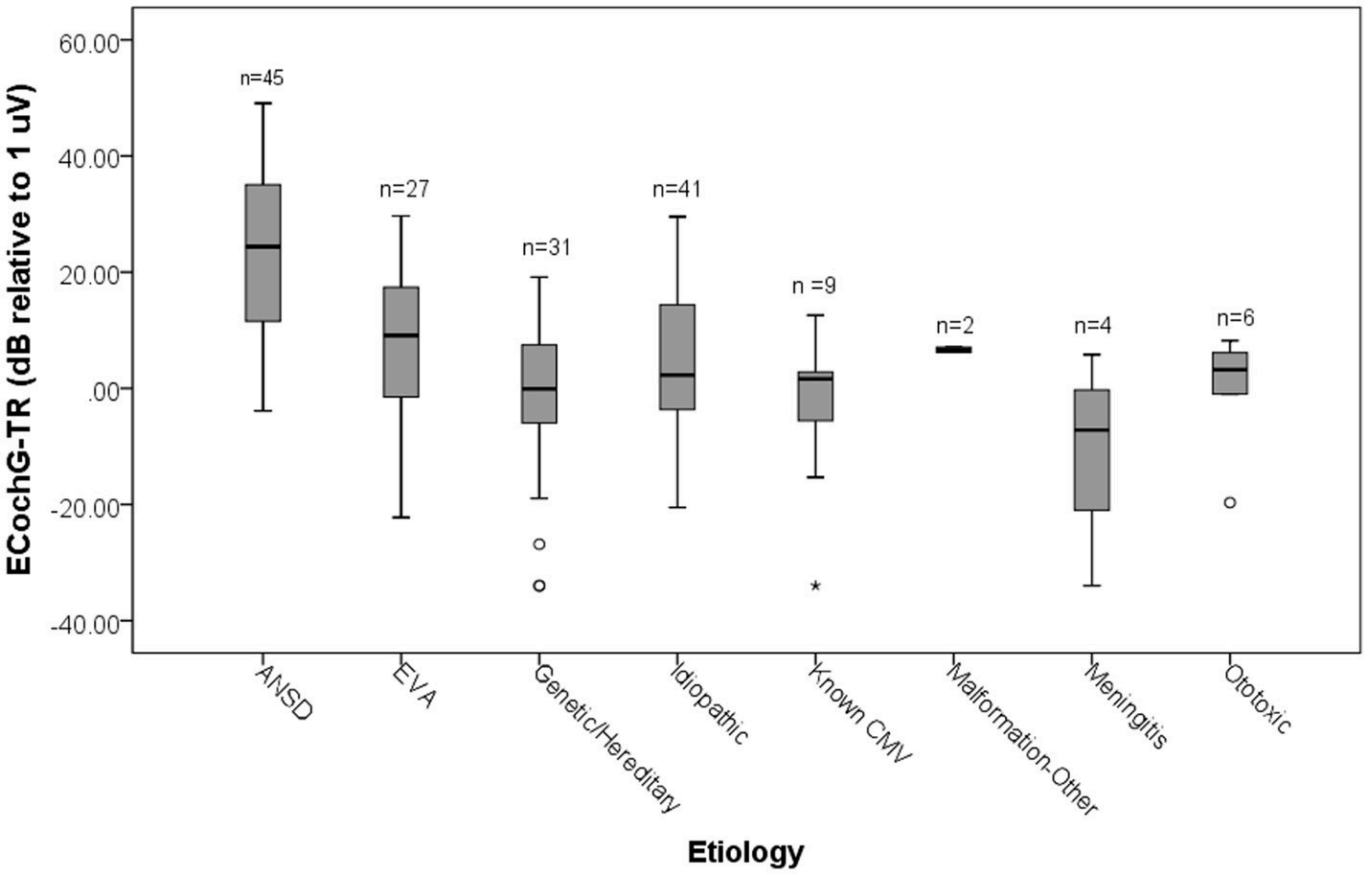
Distributions of ECochG-TR by etiology in children. ANSD subjects had the highest ECochG-TR magnitudes. Outliers: o, Close extreme; ^*^, Far extreme.

Most of the ANSD cases were children, who have a distinct mix of hearing loss etiologies that leads to CI use. As expected from the results in Figure 2, when evaluating the distribution of ECochG-TR across etiologies for the pediatric ears, the ANSD group demonstrated larger overall magnitudes compared to all other etiologies (Figure 3) The ANSD children almost universally had an ECochG-TR >1 μV (0 dB in the graph) with a mean magnitude of 23.6 ± 13.6 dB (standard deviation). A large fraction of the ANSD subjects had responses greater than 10 μV (20 dB on the graph). In contrast, for the non-ANSD etiologies a significant fraction had an ECochG-TR of <1 μV and few had values larger than 10 μV. Etiologies associated with widespread cochlear inflammation and fibrosis (meningitis and CMV) had among the lowest ECochG-TRs.

### Degrees of neural activity in ANSD subjects

**Figure 4:**
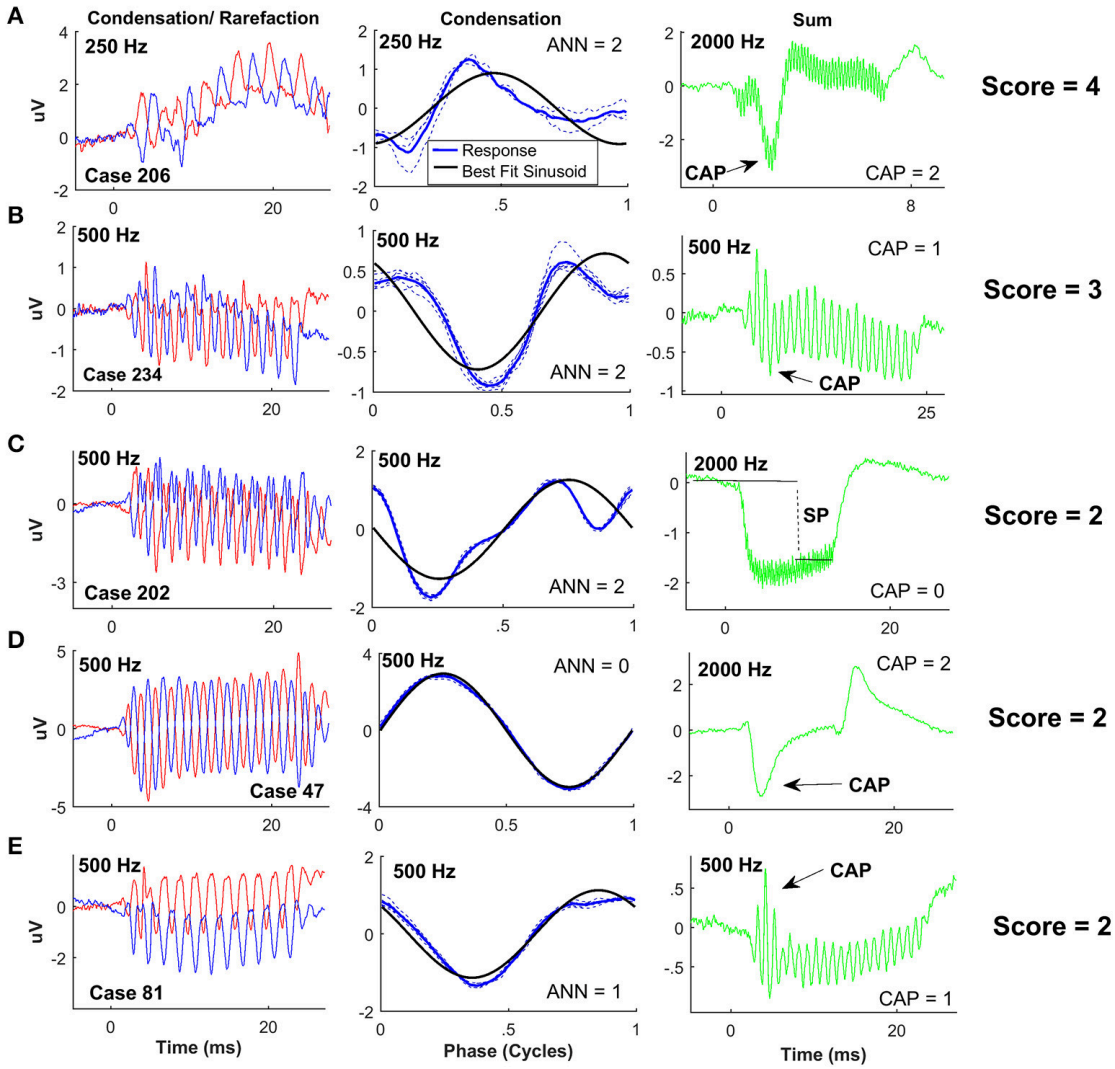
ECochG examples of ANSD subjects with considerable evidence of neural activity. For each case the response to condensation and rarefaction phase of a low frequency stimulus is shown on the left. The middle panels show the individual (dotted lines) and average cycles (thick line) to condensation phase stimuli taken from a window (8–20 ms) intended to isolate the ongoing, or steady state portion of the response. The solid black line is the best fit sinusoid. The right panels show the sum of responses to the two phases, for the frequencies as shown, which isolates the CAP and SP. **(A)** Phenotype demonstrating a score of 4, with a strong ANN shown by the distortions on the average cycles, and a strong CAP to 2,000 Hz. **(B)** This case had a nerve score of 3 with a strong ANN and small but clear CAP. **(C)** A case with a nerve score of 2, and a phenotype demonstrating a strong, ANN but no CAP, and a large negative SP. **(D)** Another case with a nerve score of two with no apparent ANN but a strong CAP. Here the SP was small. **(E)** Another nerve score of two with a phenotype showing a small CAP and ANN.

It might be expected that the large responses seen in the ANSD cohort would be associated with relatively low nerve activity. However, we found a wide spectrum of neural responses, which spanned the full range of “nerve scores.” Examples of ANSD cases with nerve scores demonstrating a high degree of neural activity, in the form of a CAP and/or ANN, are shown in Figure 4. The left panels show the summed responses to a low frequency stimulus, and the middle panels show each cycle plotted individually (dotted lines) to produce an “average cycle”(thick line). The black line in the middle panels represents the best fit sinusoid to each case, which was used for the visual analysis of the ANN (see Section Methods). The right panels of Figure 4 show the summed responses to the alternated stimulus phases. These curves emphasize the CAP which is used in the nerve score, and also help visualize the SP, which has a strong hair cell component, and will be described further in later sections.

The case in Figure 4A showed strong distortions in the ANN as well as a prominent CAP, so the ANN and CAP were both individually scored a 2 for a total nerve score of 4. The other cases (Figures 4B–E) had nerve scores of 2 or 3, derived through different combinations of the ANN and CAP, as indicated.

**Figure 5:**
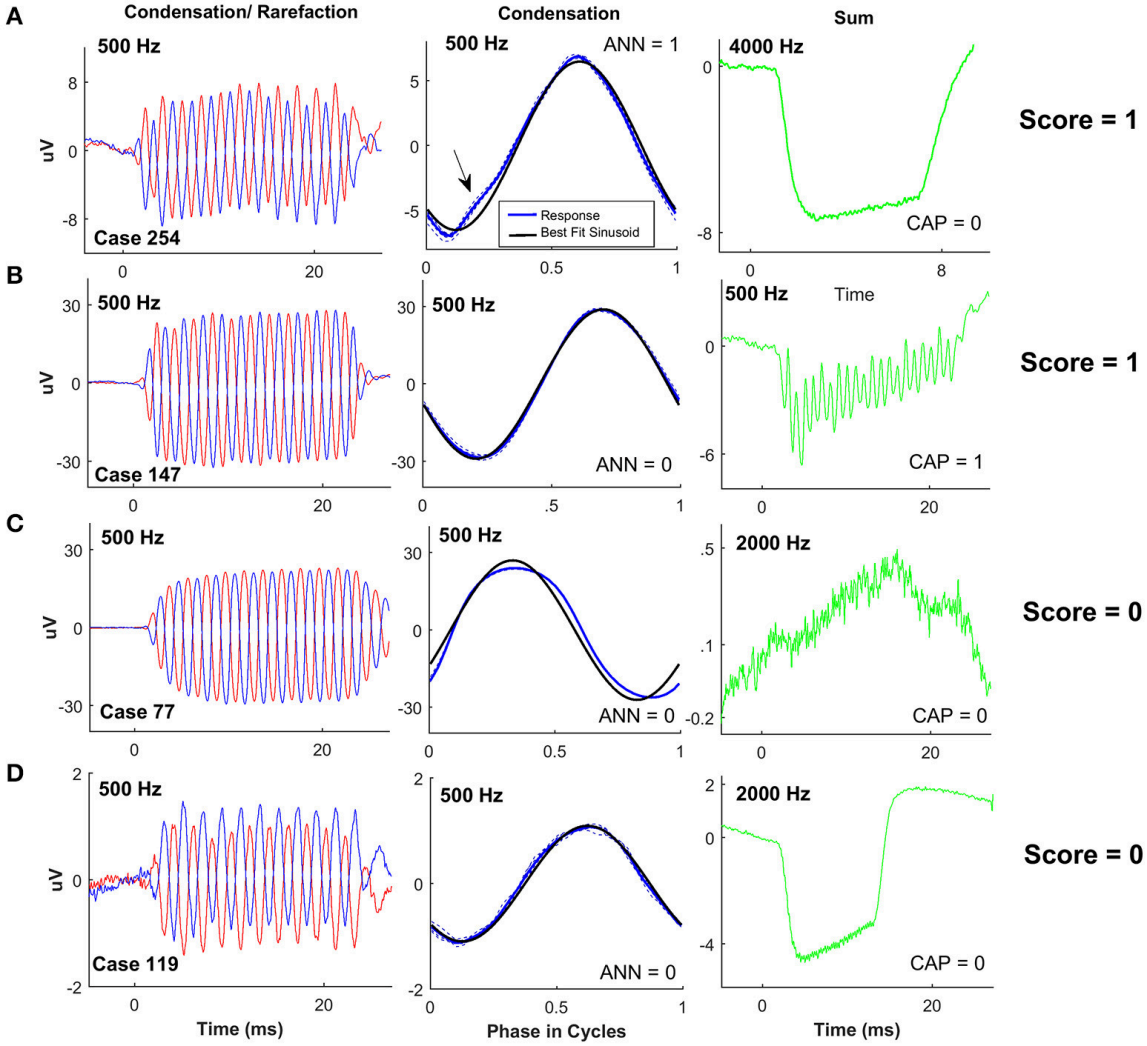
ECochG examples with minimal neural activity. **(A)** A case with a nerve score of 1, showing a small ANN (arrow) and no CAP, and a large negative SP. **(B)** Another case with a nerve score of 1, that had a phenotype with no ANN and a small CAP. **(C)** A case with no ANN or CAP, and no SP either. **(D)** Another case with no ANN or CAP, but a large negative SP.

Examples of cases with nerve scores demonstrating a low degree of neural activity (nerve score ≤ 1) are shown in Figure 5. The case in Figure 5A showed a small ANN (middle panel with arrow), and no CAP (right panel), so the nerve score was 1. The case in Figure 5B showed no ANN but a small CAP, so the nerve score was also 1. In Figures 5C,D there was no CAP or ANN, so the nerve scores were both zero. However, the SPs were markedly different in these cases.

**Figure 6:**
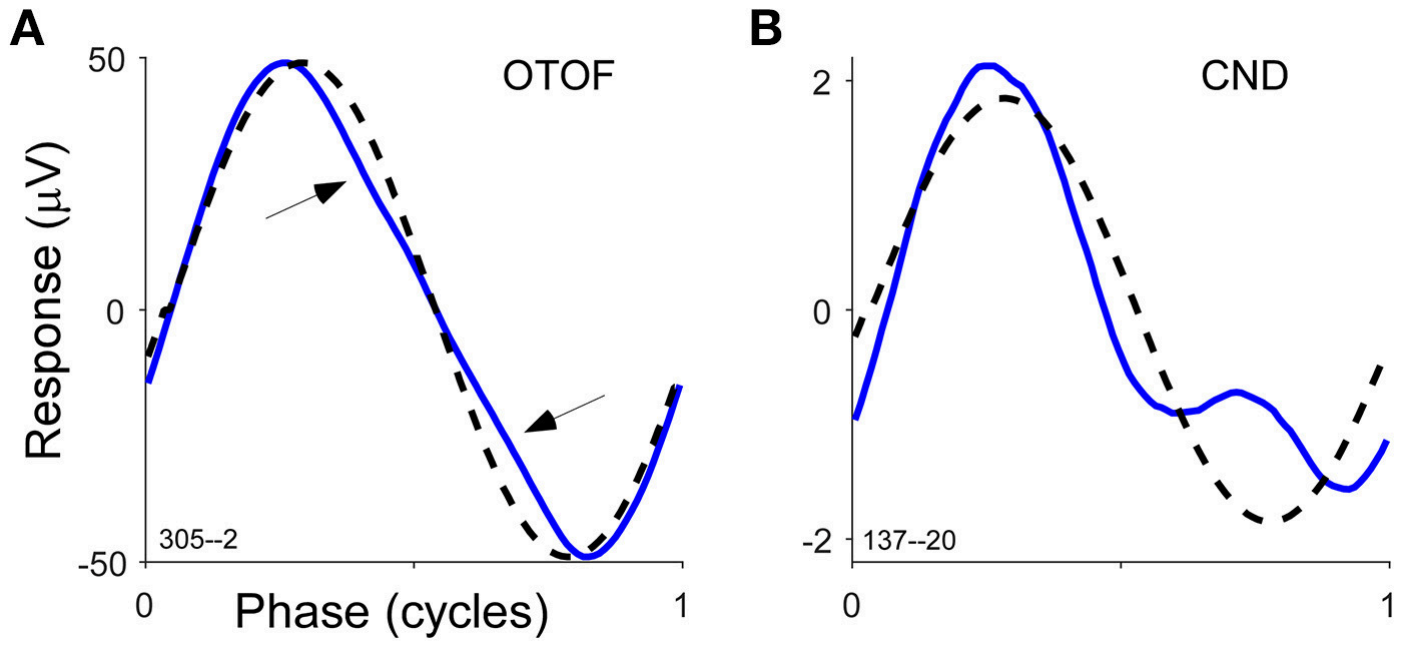
Average 500 Hz cycle for two different known etiologies of the ANSD group. **(A)** Subject had confirmed otoferlin gene mutation. To 500 Hz distortions (arrows) due to the ANN can be identified. **(B)** Subject had cochlear nerve deficiency as identified by neuroimaging but has strong ANN distortions in the 500 Hz average cycle. These examples illustrate the sensitivity of ECochG for detecting neural activity when little is expected to be present.

Two additional cases in ANSD subjects help to show the sensitivity of the method to identify neural activity even in cases where it is expected to be small. One of the cases was a 1 year old with a mutation in the gene for otoferlin, a protein required for docking of vesicles containing neurotransmitter. This was the only one of our sample with this etiology. This presynaptic site of lesion should block the ANN but not affect transduction, so the phenotype expected is a large CM with no ANN. The case did show a very large CM to all frequencies as expected. However, there was also evidence for neural activity in the average cycle to a 250 Hz tone (Figure 6A). The deviation from the sinusoid (arrows) is small, but in a signal this large all of the individual cycles lie on top of each other and each shows this same feature, so it is not attributable to noise. This type of distortion also has no clear correlate in the CM (see Figure 1A, and so instead is most likely to be due to neural activity). The second case was of cochlear nerve deficiency, and was the only one of these cases (*n* = 4) where the ANN was apparent in the average cycle to 250 (Figure 6B). These examples help to illustrate that the responses of tones to low frequencies can provide a highly sensitive means of assessing neural activity.

#### Distributions of nerve scores

The distributions of the different patterns of ANN and CAP are shown in Table 1. There was a wide spectrum of nerve scores, with scores of 2 or higher seen in 29 ears while 19 had nerve scores ≤1. An ANN score of 2 was seen in 20 ears compared to only 8 for the CAP, and the ANN scores were higher than the CAP for 23 cases compared to only 9 where the CAP had the higher score.

**Table 1:** Distribution of nerve scores in ANSD subjects.

**CAP**	**+**	**ANN**	**=**	**Nerve score**	**No. of cases**
2		2		4	6
1		2		3	12
2		1		3	0
0		2		2	2
2		0		2	2
1		1		2	7
1		0		1	7
0		1		1	9
0		0		0	3
			Total		48

**Figure 7:**
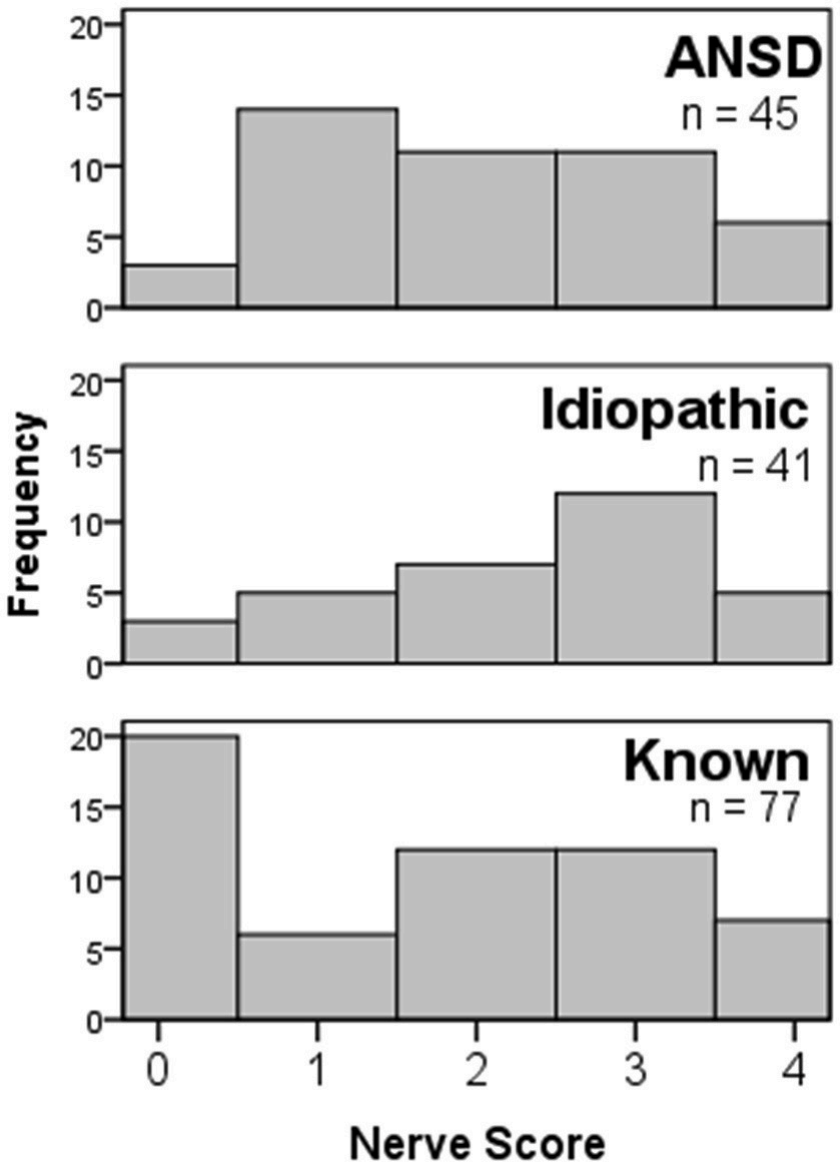
Nerve score distributions for children with different etiologies of hearing loss. All groups showed the full range of nerve scores, and the distribution in the ANSD group was not significantly different from the others.

To compare the nerve scores among the different groups, only those where the ECochG-TR was >0.5 μV were used for the non-ANSD groups. The nerve score for responses smaller than this were always 0 because components other than the CM could not be visually distinguished. All of the ANSD subjects had and ECochG-TR >0.5 μV. Nerve scores were not significantly different among subjects with different etiologies of hearing loss (Figure 7) including ANSD (Kruskal–Wallis, df = 2, chi-sq = 5.88, *p* = 0.053). The near-significant *p*-value is due to the relatively high nerve scores among subjects with idiopathic hearing loss compared to subjects with known non-ANSD etiologies. The median nerve scores among subjects with ANSD were in-between the two non-ANSD groups.

#### A negative SP and a phenomenon of “offset overshoot” may be related to a lack of neural activity

To frequencies of 1,000 Hz or greater the SP could be prominent, but to lower frequencies it was typically small. In response to the higher frequencies, three morphologies of the SP were observed, as illustrated in Figures 4, 5. One morphology was a large, negative SP (right panels in Figures 4C, 5A,D). This morphology was associated with either no CAP or only a small CAP. In cases with a large CAP (Figures 4A,D), the SP was small, and could be negative or positive. Finally, some cases had a large CM but no SP (Figure 5C). These latter two cases (Figures 5C,D) are interesting because they are both cases of cochlear nerve deficiency, and the difference in SP magnitude may be indicative of different sites of lesion (see Section Discussion).

The distributions of SP polarity and magnitude differed among ANSD and non-ANSD groups. The frequency where these differences were most clearly seen was 2,000 Hz as in Figures 4, 5. As shown in Table 2, most of the ANSD cases (17/27) had a negative SP and no CAP to 2,000 Hz. Only 3 cases had a CAP to 2,000 Hz, so in the calculation of the nerve score, most of the CAPs were seen to frequencies of 1,000 Hz and below. In contrast, cases with the features of no CAP and a negative SP were uncommon in the two non-ANSD groups (6/32 combined). The number of cases included in the two-non ANSD are relatively reduced compared to the ANSD group, because of the few cases with good responses to this high frequency (Figure 2).

**Table 2:** SP morphologies to 2,000 Hz at 90 dB nHL in ANSD and non-ANSD subjects.

**SP morphologies**	**ANSD**	**Children non-ANSD**	**Adults non-ANSD**	**Total**
No SP	7	5	8	20
CAP, small SP	3	3	10	16
Negative SP, no CAP	17	1	5	23
Total	27	9	23	59

**Figure 8:**
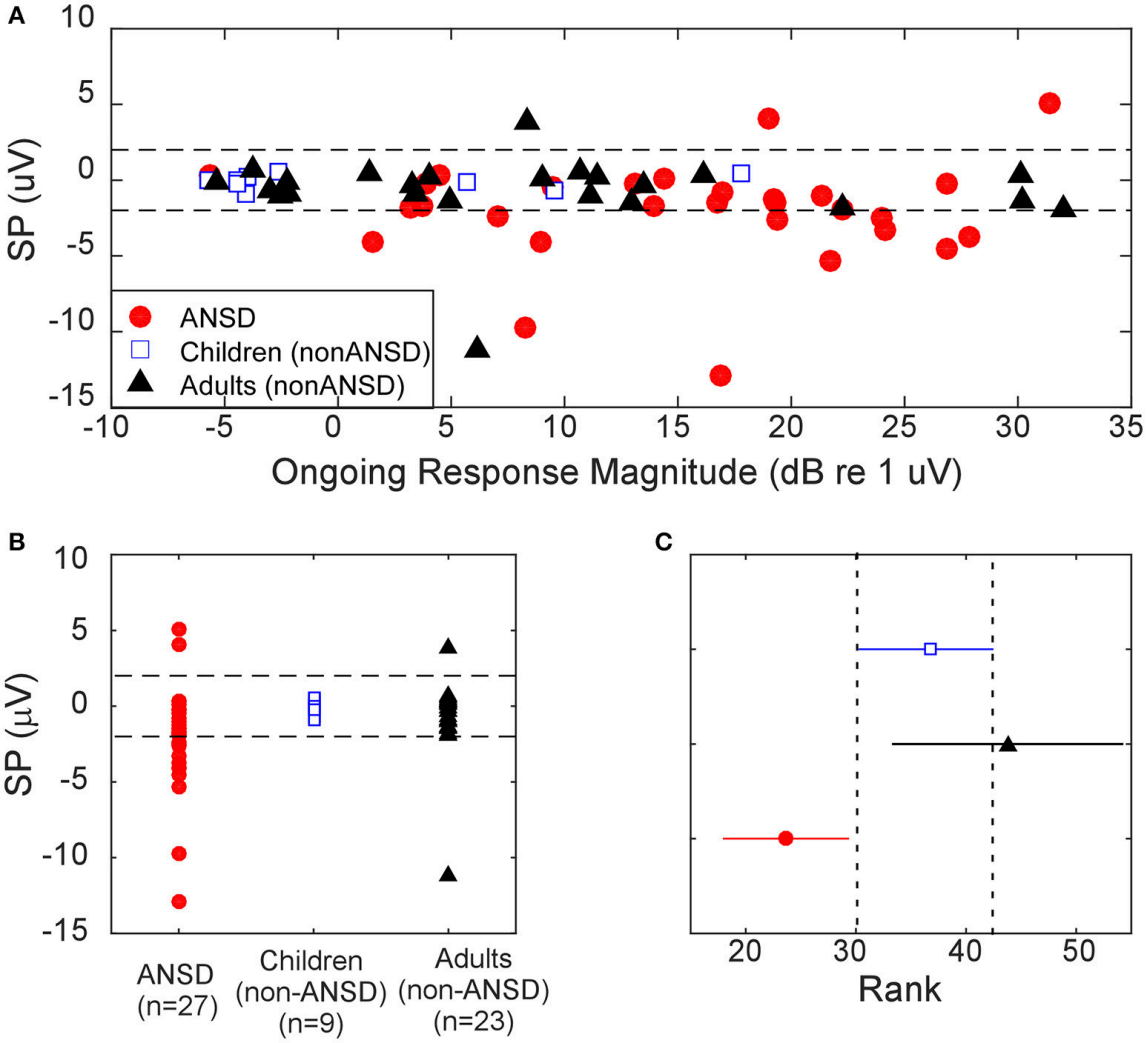
Distributions of the SP to 2,000 Hz at 90 dB nHL among the three groups. **(A)** SP distribution among the groups as a function of the ongoing response. **(B)** Distributions of the SP by group. **(C)** Mean SP rank by group. Dotted lines are the 95% confidence interval around the non-ANSD children.

Illustration of the differences in the values of SP for ANSD and non-ANSD subjects is shown in Figure 8. In Figure 8, the magnitude and polarity of the SP to 2,000 Hz at 90 dB nHL are plotted against the magnitude of the ongoing response. The dotted lines are shown at ±2 μV, to highlight that most of the cases with negative SPs were ANSD subjects, while most non-ANSD cases had SPs near zero. One ANSD and one adult non-ANSD case had positive SPs >2 μV. The ANSD case had no CAP and the morphology of the SP was similar to those with negative SPs, but reversed. The non-ANSD case with a large, positive SP had a large CAP. The distributions of the SP values are shown in Figure 7B. There was a main effect of group (Kruskal–Wallis test, dfs = 2, chi-sq = 11.4, *p* = 0.003) and multiple comparisons of the mean ranks showed the ANSD group to have a significantly more negative SP overall compared to the other groups, which did not differ between themselves (Figure 8C).

**Figure 9:**
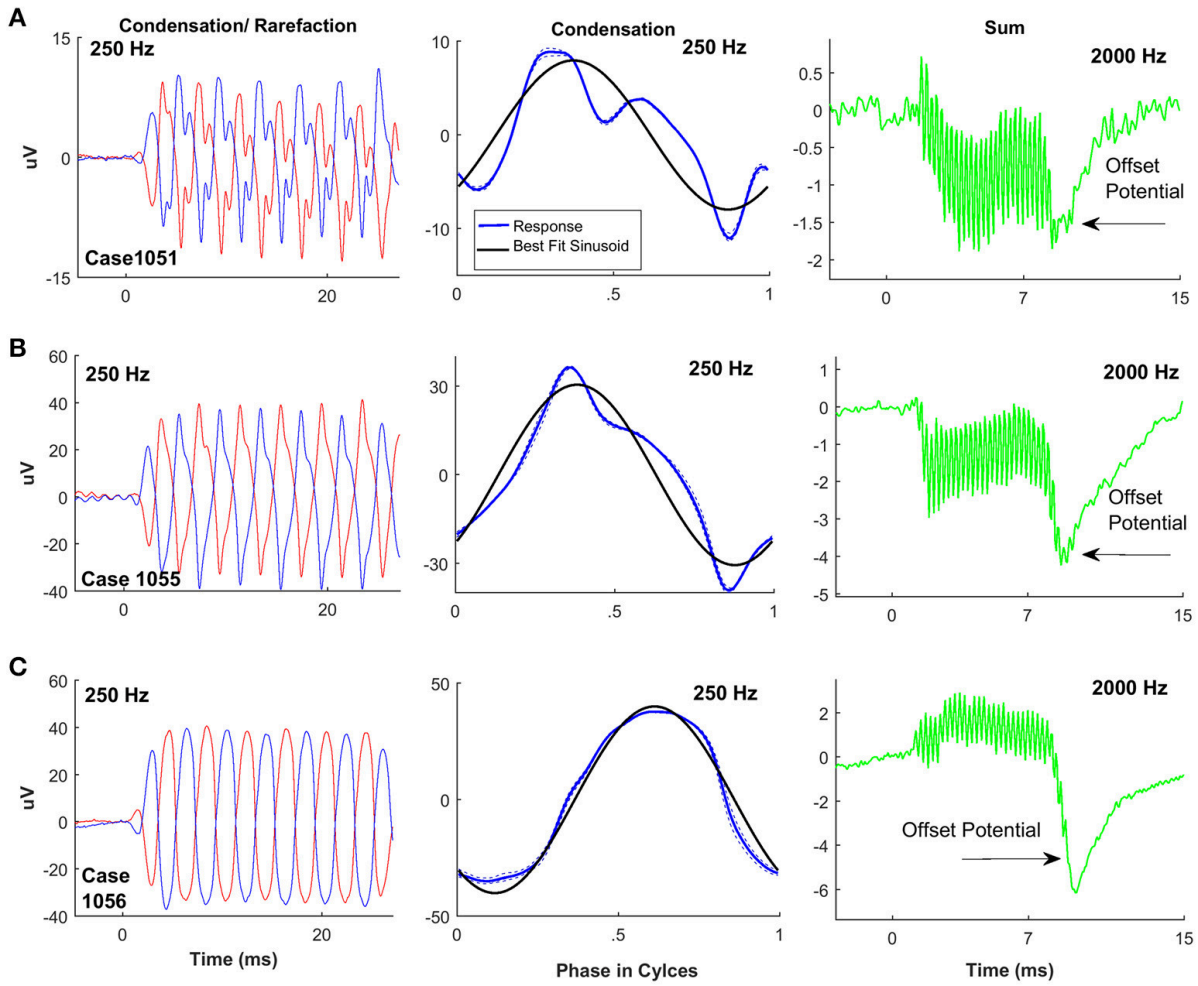
Overshoot potential in some ANSD children reflected in the “sum” tracing. **(A–C)** Three different cases showing the offset overshoot. Note that there is no onset CAP, so this overshoot is unlikely to be related to a CAP to the offset.

In addition to the SP, a number of ANSD cases (*n* = 5) were seen which demonstrated an offset overshoot to 2,000 Hz (Figures 9A–C, right panel). No identifiable onset CAP was discernable in any of the ears where this overshoot was observed. In addition, a similar overshoot is often seen in gerbil responses after a neurotoxin has been applied (personal observations). Tentatively therefore, we consider this overshoot to be related to the SP. The SP is a complex mixture of sources with different polarities and time courses, so complex phenomena can be expected under different hearing conditions.

### Adult cohort

Three adults with ANSD were identified by the presence of CM on ABR, after audiological testing had suggested ANSD. As with the children, the ECochG-TRs in adults were large (Figure 2) and the degree of neural activity varied considerably even in this small group, with nerve scores of 1–3, demonstrating a mix of ANN and CAP involvement.

## Discussion

Our expectation was that ANSD subjects would have a large cochlear response and relatively little neural activity compared to other CI subjects. The results were that ANSD cases had on average a larger ECochG-TR; the responses extended more often to high frequencies; and responses to each frequency were on average larger in ANSD compared to non-ANSD cases. However, in the ANSD cases there was a full range of “nerve scores,” derived from CAPs and the ANN, with the scores dominated by the presence of the ANN. Thus, to low frequencies there was little difference in neural activity in ANSD compared to non-ANSD cases. In contrast, to high frequencies the majority of ANSD cases showed no CAP and a strongly negative SP, while this pattern was rare in the non-ANSD groups. Thus, the hallmark of ECochG in subjects with a clinical report of ANSD is of large responses with a lack of neural activity to high frequencies, combined with responses to low frequencies that have the same distribution of neural activity as found in non-ANSD cases. In the following, we will describe how these attributes are fully compatible with the main clinical findings of a CM with small or absent wave V in ABR results.

### The cochlear microphonic in ANSD subjects

To high frequency stimuli the cyclic response to tones consists purely of the CM, since it is above the range of phase-locking in the auditory nerve. The main distinction of ANSD compared to other CI subjects is the large CM to high frequencies, which accounts for the appearance of the CM in ABR recordings from these subjects. We did not fully explore the upper end of the frequency range, since in most subjects the highest frequency used was 4 kHz, where most ANSD subjects still had robust responses, in contrast to the non-ANSD groups, where responses to 2 and 4 kHz were relatively rare.

To low frequencies the responses in ECochG are still primarily the CM, even though they can be mixed with the ANN, when present. Thus, the larger overall responses to low frequencies in ANSD compared to non-ANSD subjects could indicate greater CM from the apex than in the non-ANSD groups. However, a more likely cause is the additional CM from higher CF regions of the cochlea that respond to low frequency stimuli as well.

The presence of the CM indicates the integrity of hair cells, but it cannot be specifically localized to outer hair cells, as is generally understood to be the case in studies of normal hearing animals (Dallos, 1973). This determination is difficult because both inner and outer hair cells produce a CM, and the pattern of hair cell loss in an individual subject is unknown. The presence of OAEs would be a more direct measure of functional outer hair cells, but a CM could be derived from the low CF cochlear regions where OAEs are not tested, and/or damaged hair cells that generate a CM but do not produce a functioning cochlear amplifier. Other responses features, in particular the SP, might be able to contribute to the determination of OHC vs. IHC activity.

### Neural activity in ECochG: the compound action potential and ANN in ANSD subjects

The CAP is a highly variable feature in CI subjects (Scott et al., 2016), including those with ANSD, as documented here. When present, it is a clear indication of neural activity. However, its absence does not fully assess neural activity, since the ANN was more prevalent than the CAP. We tried numerous methods to quantify the ANN prior to adopting the subjective method ultimately used. The ANN contributes to a 2nd harmonic in the response (Henry, 1995; Lichtenhan et al., 2013; Forgues et al., 2014), but the amount of the 2nd harmonic is not directly related to the size of the ANN because (1) most of its energy is at the first harmonic, where it is mixed with energy from the CM, (2) phase relationships between the ANN and CM can cause the net magnitudes in each harmonic to vary independent of their strength, and (3) the CM can produce harmonics of both even and odd order as well, so the simple presence of distortions in the spectrum is not a reliable indication of the ANN. Instead, the shape of the distortions in the average cycle must be examined to determine if the harmonics could plausibly be attributed to hair cells. In addition to harmonic analysis we have tried a number of different measurements to quantify the strength of the neural activity in our responses, such as correlation with a sine wave or power-line analysis such as form factor and crest factor. Unfortunately, none has proven adequate to capture the variety of responses seen. Our qualitative approach was therefore to note the presence of the ANN in the shape of the cyclic waveform, and to estimate its strength over a narrow range. We are currently investigating modeling methods to quantify the relative contribution of the CM and ANN.

The finding of a large degree of neural activity to low frequencies seems at odds with the clinical understanding of ANSD as representing an underlying etiology that affects the chain from IHCs to the CNS differently than in non-ANSD cases. However, the main difference between the clinical definitions of ANSD used here is the presence of a CM; both groups are receiving a CI and thus have a small or absent wave V. So, as previously discussed, the presence of a CM is well accounted for by the ECochG results showing greater hair cell activity to high frequencies, and the small but measurable neural activity primarily to low frequencies across all groups accounts for the reduced magnitudes of later waves in the ABR.

### The SP in ANSD subjects

Despite its first description in the 1950s (Davis et al., 1950, 1958), the origin of the SP is still a matter of considerable debate in terms of contributions from inner and outer hair cells and neural sources. Early work suggested outer hair cell sources predominate (Dallos and Cheatham, 1976) but later studies that removed inner hair cells in chinchillas showed a large effect on the CM (Zheng et al., 1997; Durrant et al., 1998). Furthermore, animal work in gerbils using the neurotoxin kainic acid recently showed a neural contribution to the SP (Forgues et al., 2014), which had also been reported previously using other species and compounds for blocking neural activity (van Emst et al., 1995; Sellick et al., 2003). In addition to the complexity of sources, the geometry between sources and recording sites will affect the polarity of the SP, contributing to complex changes across frequency and intensity as sites of generation within the cochlea shift. With these caveats, the phenotype of a large, negative SP (positive in one case) was correlated with the absence of a CAP, and therefore presumably of sustained neural activity as well. In ANSD subjects this phenotype predominated, while it was uncommon in the other groups. Furthermore, in both ANSD and non-ANSD groups, when a large CAP did exist the morphology of the SP was distinctly different, being close to zero in most cases with no preference for polarity. These findings of a relatively reduced CAP and enhanced SP closely parallel those reported previously for ANSD subjects using high frequency stimuli such as clicks and 8 kHz tone bursts (McMahon et al., 2008; Santarelli et al., 2008; Stuermer et al., 2015).

Two cases with cochlear nerve deficiency, an extreme example of ANSD (Figures 5C,D), had distinctly different SPs, that may, or may not, be related to different sites of lesion. Both cases had large CMs and no evident neural activity, but one case had no SP, while the other had a large negative SP. The presence of the large, negative SP typical of this and other ANSD cases may be due to the presence of IHCs, which are thought to have much more asymmetrical operating point, or proportion of open channels at rest, than OHCs (Russell, 2008). Thus, the presence of the negative SP could indicate the presences of functioning IHCs, and the lack of the negative SP, combined with no neural activity, could indicate the lack of IHCs. Alternatively, however, the operating point in the IHCs and OHCs in given case may be less asymmetric than in other cases, or the “effective intensity” of the stimulus in the face of hearing loss may produce basilar membrane movement too small for any asymmetry to be evident in the ECochG. Finally, the SP in one case and not the other could be due to presence of currents related to the dendritic potential, or the sum of excitatory post synaptic currents from the terminals of auditory nerve dendrites. These possibilities show that the SP could reveal considerable insights regarding sources of residual physiology in individual cases, as its sources become better understood.

In some cases, a large transient potential was observed to the stimulus offset to high frequencies. There was no CAP at stimulus onset in these cases, so the offset potential is unlikely to be a CAP to the offset. These responses were scored as “no SP” but a small or absent SP can also indicate a balance of contributions from outer hair cells, inner hair cells, and the auditory nerve. That is, different sources of sustained potentials can sum to be near zero at the steady state, while different time courses for each source allow them to be revealed when the stimulus changes.

### Current study in relation to previous studies of ECochG in ANSD children

Most previous studies of ECochG in ANSD children undergoing cochlear implantation used 8 kHz tone pips or clicks as stimuli (Gibson and Sanli, 2007; McMahon et al., 2008; Santarelli et al., 2008; Stuermer et al., 2015). These are primarily high frequency stimuli, which is an appropriate choice for many ANSD subjects who typically have good responses to high frequencies. However, to characterize ANSD subjects in the context of the general pediatric population, tone bursts that can transmit concentrated energy to low frequencies are needed because many CI subjects have no residual responses to high frequencies. Nearly all subjects, adult and pediatric, show responses to low frequency tone bursts with high signal to noise ratio when recorded at the RW (Figure 2 and Choudhury et al., 2012; Fitzpatrick et al., 2014; McClellan et al., 2014; Dalbert et al., 2015).

The focus of much of the previous work with ECochG in ANSD subjects has been to identify phenotypes showing different sites of lesion that may result in different speech perception outcomes (Gibson and Sanli, 2007; McMahon et al., 2008). Sites can be identified as pre-synaptic by the absence of dendritic or spiking nerve activity and post-synaptic if either of these exist (McMahon et al., 2008). The idea is that if the lesion is presynaptic there is insufficient neurotransmitter release and thus a lack of neural spiking, but if there is spiking the lesion must be post-synaptic, e.g., demyelination causing asynchrony, central deficits, or loss of a fraction of synaptic connections due to excitotoxicity at the nerve terminal. Results in the current study showed that only a small number of ANSD cases did not demonstrate any evidence of a CAP or ANN, and hence had no evidence of neural spiking activity. However, rather than interpreting all of the cases with spiking activity as “post-synaptic” we think it is likely that in many instances there are still some residual neural connections primarily in low frequency regions of the cochlea, even in cases that should be considered a pre-synaptic etiology, such as otoferlin (see Figure 6). In general, therefore, the presence of neural activity in the ECochG does not necessarily indicate a post-synaptic site of lesion.

### Non-ANSD and unknown etiologies

In both adult and pediatric non-ANSD cases the ECochG-TR was on average lower than in ANSD cases. In children, those with inflammatory reactions including CMV or meningitis had the lowest ECochG-TR. For those with the smallest responses there were no detectable CAPs, ANNs or SPs that could be distinguished from a sinusoidal CM. However, to the majority of cases where these additional potentials could be detected, the neural involvement covered the full spectrum of nerve scores, similar to the ANSD group. Previously, it was noted that adults and children had similar ranges of ECochG-TR, and a similar distribution of frequencies that contributed to the responses, as also reported here in Figure 2 (Fitzpatrick et al., 2014). However, here we report that in children the upper end of the ECochG-TR distribution is mostly filled by ANSD cases, which represents a large difference from adults, in whom ANSD is uncommon.

## Conclusions

The difference between ANSD and non-ANSD subjects lies primarily in the high frequency regions of the cochlea. These regions produce a larger CM and SP, and are less likely to produce a CAP, compared to non-ANSD subjects. These features are consistent with a large hair cell response combined with a limited neural response expected for ANSD. In contrast, for responses to low frequencies the neural components, primarily in the form of the ANN, are similar between ANSD and non-ANSD subjects, and vary from no evidence of neural contributions to clear evidence of CAP and/or ANN. Therefore, responses from low frequency parts of the cochlea produce a similarly wide distribution of evidence for neural activity between ANSD and non-ANSD subjects. It remains to be determined if the levels of neural activity seen using acoustic stimuli by ECochG are important in speech perception outcomes with the CIs.

## Ethics statement

This study was carried out in accordance with the approved protocols and recommendations of the University of North Carolina at Chapel Hill's Institutional Review Board and the Ohio State University's Institutional Review Board (#05-2616 and #2015H0045) with written informed consent from all subjects. All subjects gave written informed consent in accordance with the Declaration of Helsinki.

## Author contributions

WR, JR, CG, MH, ZB, TF, CB, KB, DF, OA contributed to data collection and manuscript preparation. WR, JR, DF, and OA contributed to analysis of data.

### Conflict of interest statement

The authors declare that the research was conducted in the absence of any commercial or financial relationships that could be construed as a potential conflict of interest.
